# The Fungicide Tetramethylthiuram Disulfide Negatively Affects Plant Cell Walls, Infection Thread Walls, and Symbiosomes in Pea (*Pisum sativum* L.) Symbiotic Nodules

**DOI:** 10.3390/plants9111488

**Published:** 2020-11-04

**Authors:** Artemii P. Gorshkov, Anna V. Tsyganova, Maxim G. Vorobiev, Viktor E. Tsyganov

**Affiliations:** 1 Laboratory of Molecular and Cell Biology, All-Russia Research Institute for Agricultural Microbiology, 196608 Saint Petersburg, Russia; artemius1993@yandex.ru (A.P.G.); vetsyganov@arriam.ru (V.E.T.); 2 Research Resource Centre “Molecular and Cell Technologies”, Saint Petersburg State University, 199034 Saint Petersburg, Russia; vorobiev.maxim@spbu.ru; 3 Saint Petersburg Scientific Center RAS, 199034 Saint Petersburg, Russia

**Keywords:** *Pisum sativum* L., symbiotic nodule, infection thread, symbiosome, bacteroid, cell wall, fungicide

## Abstract

In Russia, tetramethylthiuram disulfide (TMTD) is a fungicide widely used in the cultivation of legumes, including the pea (*Pisum sativum*). Application of TMTD can negatively affect nodulation; nevertheless, its effect on the histological and ultrastructural organization of nodules has not previously been investigated. In this study, the effect of TMTD at three concentrations (0.4, 4, and 8 g/kg) on nodule development in three pea genotypes (laboratory lines Sprint-2 and SGE, and cultivar ‘Finale’) was examined. In SGE, TMTD at 0.4 g/kg reduced the nodule number and shoot and root fresh weights. Treatment with TMTD at 8 g/kg changed the nodule color from pink to green, indicative of nodule senescence. Light and transmission electron microscopy analyses revealed negative effects of TMTD on nodule structure in each genotype. ‘Finale’ was the most sensitive cultivar to TMTD and Sprint-2 was the most tolerant. The negative effects of TMTD on nodules included the appearance of a senescence zone, starch accumulation, swelling of cell walls accompanied by a loss of electron density, thickening of the infection thread walls, symbiosome fusion, and bacteroid degradation. These results demonstrate how TMTD adversely affects nodules in the pea and will be useful for developing strategies to optimize fungicide use on legume crops.

## 1. Introduction

Legumes, including peas (*Pisum sativum* L.), are important food and feed crops. In 2018, the largest pea producers in the world were Canada (3.6 million tonnes), Russia (2.3 million tonnes), and China (1.5 million tonnes) [1]. Forecasts by Mordor Intelligence predict world pea production to grow by an average of 5.9% from 2019 to 2024 [2]. Peas are suitable for cultivation in regions with cool climates covering more than 12 million ha in more than 100 countries worldwide. Dry pea production is ranked fourth in the world among legumes, after soybeans, peanuts, and dry beans [3]. In Russia, pea crops occupy about 70% of the area under grain legume cultivation.

The pea is affected by many types of fungal and bacterial diseases, the most harmful of which are root rots, damping-off diseases, and rusts [4]. Powdery mildew, bacterial wilts, and blights frequently cause severe damage to crops [5,6]. Currently, *Fusarium* wilt and damping-off are the most prevalent diseases in Russia, and result in crop losses of 30–50% or more. In some years, entire crops are lost to these diseases [7]. Fungicide application and agronomic practices are proposed as control measures to reduce the severity of disease, minimize crop losses, and increase the contribution of crops to food security [5,8,9].

In many countries, including Russia, a fungicide commonly used to treat seeds of the garden pea is tetramethylthiuram disulfide (or thiram; TMTD). It is a protective contact fungicide that does not penetrate plant tissues or seeds and inhibits spore germination or the initial growth of surface pathogenic mycelium [10,11]. This compound actively suppresses the development of pathogenic oomycetes and partially inhibits growth of basidiomycetes. After penetrating the cells of the pathogen, TMTD inhibits the activity of enzymes containing copper or sulfhydryl groups [12]. On treated grains in the soil, TMTD retains its fungicidal activity for up to 30 days. In plants and soil, TMTD decomposes to the more toxic and environmentally hazardous metabolites tetramethylthiuram monosulfide and tetramethylthiourea [10,12]. Among synthetic fungicides, TMTD is the most suitable for treating legume seeds because of its low toxicity to certain types of rhizobia.

Similar to other legumes, the garden pea enters symbiotic relationships with rhizobial soil bacteria to form symbiotic nodules, allowing it to fix nitrogen from the atmosphere. Fungicides have the strongest inhibitory effect on soil microorganisms [13], and most fungicides are also toxic to rhizobia. Inhibition of rhizobia can lead to disruption of nodulation or a decrease in nitrogen fixation. Therefore, the effects of fungicide treatment should be considered from the perspective of the legume–rhizobial symbiosis [14].

Previous studies have explored the diverse impacts of pesticides on the legume–rhizobial symbiosis. These effects vary depending on the selected pesticide and its concentration [15,16,17], and the rhizobial species [18] or strain [19,20]. Detrimental effects of fungicides on rhizobia in vitro have been reported [15,21]. Several studies have shown that exposure to insecticides and TMTD inhibits the growth of various strains of *Bradyrhizobium japonicum* cultures [15,22,23]. When applied alone or in combination with insecticides, TMTD significantly inhibits plant growth and influences nitrogenase activity during nitrogen fixation [24]. However, it has a negligible effect on the growth of *Rhizobium leguminosarum* bv *phaseoli* [23].

Previous attempts to isolate fungicide-insensitive rhizobia strains for seed inoculation have resulted in the isolation of *R. meliloti* [25], *R. phaseoli* [26], and *B. japonicum* [21]. However, strains that show intrinsic resistance to one fungicide can be acclimatized only to that fungicide, but not to others.

The effect of TMTD on nodule formation and nitrogen fixation depends not only on the rhizobial species, but also on the plant species. Different fungicides vary in their effects depending on the symbiotic system involved. An evaluation of the effect of a variety of fungicides applied as a seed treatment to white clover (*Trifolium repens* L.), pea, and soybean (*Glycine max* (L.) Merr.) revealed that TMTD and rhizoctol are harmful to clover and the pea [27]. In peas treated with TMTD, nitrogenase activity decreases, the size, weight, and number of nodules decreases significantly, and white ineffective nodules are formed [28]. Treatment of pea and lentil (*Lens culinaris* Medik.) seeds with TMTD reduces the incidence of seedling damping-off caused by *Pythium* sp., improves plant growth, and increases seed yield [29]. In chickpea (*Cicer arietinum* L.), low concentrations of TMTD increase yields [30]. However, in field experiments, TMTD was found to decrease the productivity of a *Mesorhizobium ciceri* strain, leading to decreased root and shoot biomass and seed yield of chickpea [31]. Nodulation of TMTD-treated soybean plants is significantly reduced compared with that of untreated plants, while the nodulation of bean (*Phaseolus vulgaris* L.) is unaffected by TMTD treatment [23,32].

Thus, previous studies have reported contradictory data for the effects of TMTD on nodulation. The effect of TMTD depends on many components, such as the rhizobial species and strain, the period of contact with the fungicide and its concentration, and many environmental variables. The aim of the present study was to determine the effect of high concentrations of TMTD applied directly to the substrate on the structure of pea symbiotic nodules. The data obtained indicate an adverse effect of TMTD on plant cells in symbiotic nodules.

## 2. Results

### 2.1. Nodulation and Plant Growth Parameters

**Figure 1:**
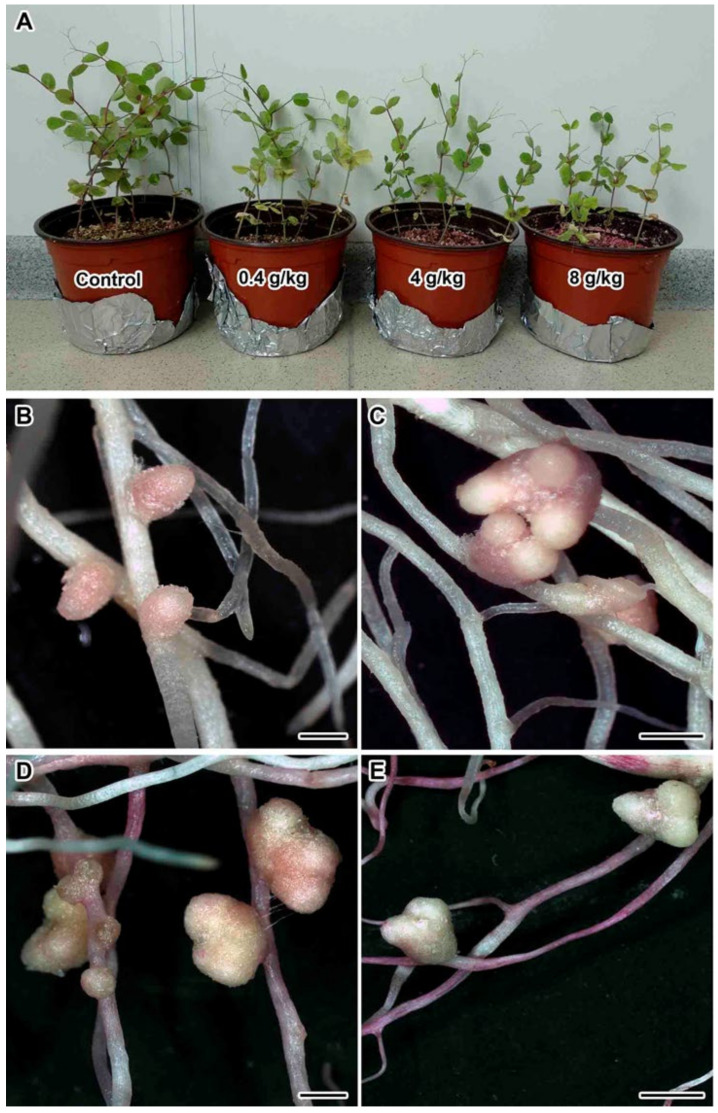
Phenotypes of plants and nodules of pea (*Pisum sativum* L.) laboratory line SGE treated with tetramethylthiuram disulfide (TMTD). (**A**) Phenotype of plants treated with 0.4, 4, or 8 g/kg of TMTD. (**B**) Control roots and nodules (no fungicide treatment). (**C**) Roots and nodules of plants treated with 0.4 g/kg of TMTD. (**D**) Roots and nodules of plants treated with 4 g/kg of TMTD. (**E**) Roots and nodules of plants treated with 8 g/kg of TMTD. Scale bar (**B**–**E**) = 1 mm.

**Figure 2:**
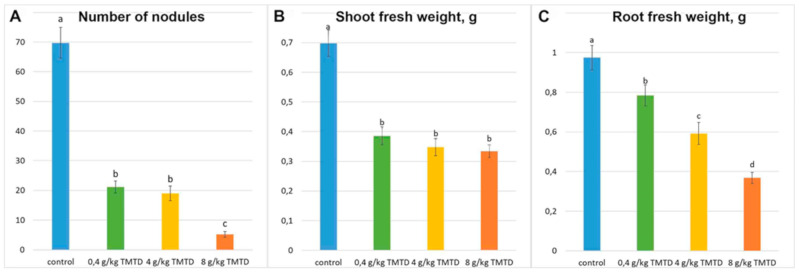
Influence of TMTD concentration on growth parameters of pea (*Pisum sativum* L.) laboratory line SGE. (**A**) Mean nodule number per plant. (**B**) Mean shoot fresh weight. (**C**) Mean root fresh weight. Different letters indicate significant difference according to the least significant difference test (*p* < 0.05; *n* = 15).

Treatment with TMTD affected plant size and nodule morphology in the three studied pea genotypes. A detailed phenotypic analysis was performed using the laboratory pea line SGE. With the increase in TMTD concentration, the shoot height decreased (Figure 1A), the shoot width decreased, the leaves yellowed, and accumulation of fresh weight (FW) significantly decreased (Figure 2B,C). In addition, TMTD negatively affected root development. In response to 8 g/kg of TMTD, the length and width of the main and lateral roots were reduced (Figure 1E). Treatment with 4 g/kg of TMTD caused similar, but less pronounced, phenotypic changes (Figure 1D). As with the shoot FW, root FW significantly decreased with the increase in TMTD concentration (Figure 2C). Pearson’s correlation analysis revealed significant negative correlations between TMTD concentration and shoot and root fresh weights, and a significant positive correlation between shoot and root fresh weights. The shoot and root fresh weights showed relatively high dose dependency with respect to TMTD (Table 1).

Nodules formed in each treatment (Figure 1B–E), but significantly fewer formed on TMTD-treated plants than on untreated (control) plants (Figure 2A). Nodules with multiple meristems formed in response to TMTD treatment (Figure 1C–E). In response to 4 g/kg of TMTD, pale nodules with a green base formed, which may indicate the induction of nodule senescence (Figure 1D). Under treatment with 8 g/kg of TMTD, most nodules were green (Figure 1E), and the fewest nodules formed in this treatment (Figure 2A). Pearson’s correlation analysis revealed significant positive correlations between the number of nodules and shoot and root fresh weights. In addition, the TMTD concentration was significantly negatively correlated with nodule number. Thus, an increase in the TMTD concentration corresponded to a significant decrease in nodule number (Table 1).

### 2.2. Light Microscopy Analyses

**Figure 3:**
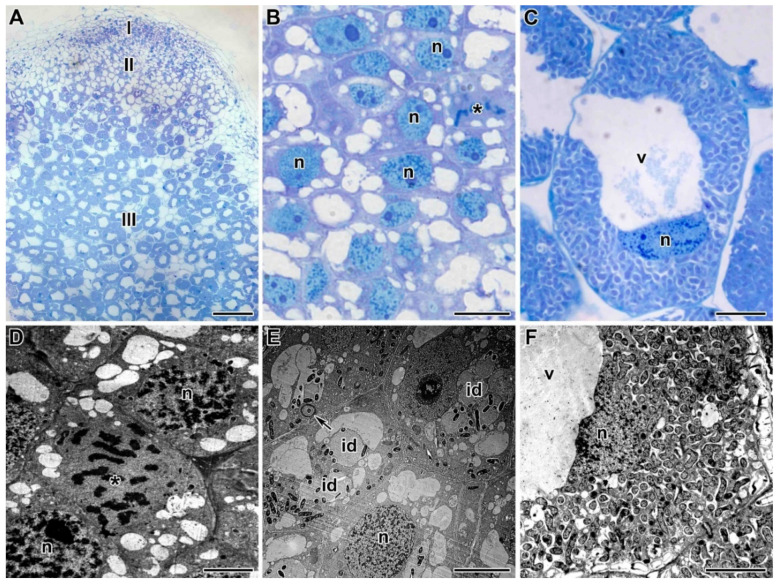
Histological and ultrastructural organization of untreated nodules from 3-week-old plants of three pea genotypes. (**A**) Longitudinal section of a nodule of laboratory pea line SGE. (**B**,**D**) Meristematic cells of a nodule of laboratory pea line SGE. (**C**) Infected cells in nitrogen-fixation zone of a nodule of laboratory pea line SGE. (**E**) Cells in the infection zone of a nodule of cultivar ‘Finale’. (**F**) Infected cells in nitrogen-fixation zone of a nodule of laboratory line Sprint-2. **I**—meristem, **II**—infection zone, **III**—nitrogen fixation zone, **n**—nucleus, **id**—infection droplet, **v**—vacuole, *—metaphase plate. Arrows indicate infection thread. Bars (**A**) = 50 µm, (**B**–**F**) = 5 µm.

The light microscopy analyses showed that the control pea root nodules had a typical elongated shape and structure typical of an indeterminate nodule with characteristic zonation (Figure 3A). In three-week-old pea wild-type nodules, a meristem consisting of dividing cells, an infection zone, and a nitrogen-fixation zone could be distinguished. Meristematic cells had numerous small vacuoles, a large nucleus with a nucleolus, and an electron-dense cytoplasm (Figure 3B). Metaphase plates were often visible. Numerous infection threads and droplets were present in the infection zone, and a few juvenile bacteroids were located along the cell periphery (data not shown). Mature nitrogen-fixing cells with the central vacuole were filled with numerous pleiomorphic bacteroids (Figure 3C).

**Figure 4:**
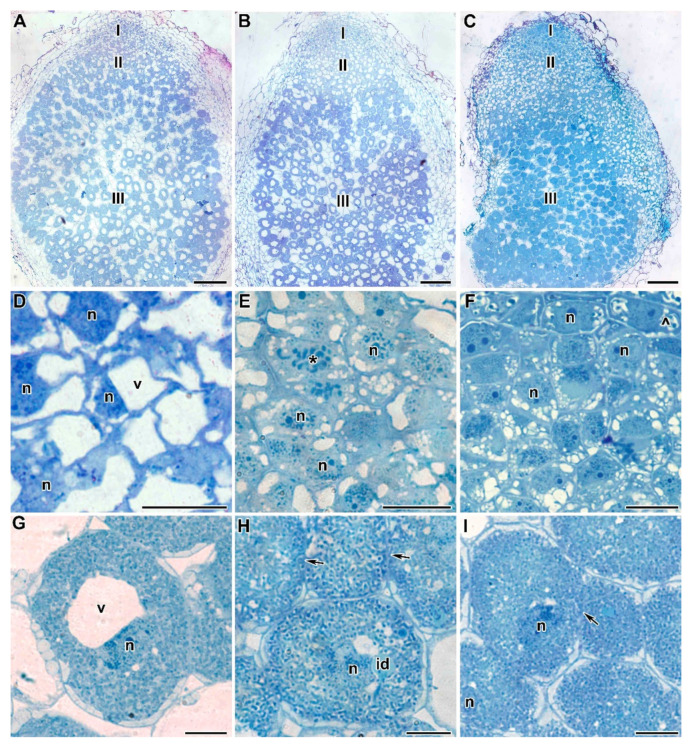
Light microscopy micrographs of 3-week-old plants of three pea genotypes treated with 0.4 g/kg of TMTD. (**A**–**C**) Longitudinal sections of 3-week-old pea nodules. (**D**–**F**) Meristem of pea nodules. (**G**–**I**) Infected cells in the nitrogen-fixation zone of a nodule. Laboratory line Sprint-2 (**A**,**D**,**G**), laboratory line SGE (**B**,**E**,**H**), cultivar ‘Finale’ (**C**,**F**,**I**). **I**—meristem, **II**—infection zone, **III**—nitrogen-fixation zone, **n**—nucleus, **id**—infection droplet, **v**—vacuole, *—metaphase plate, **^**—vacuole inclusions, arrows indicate the barely distinguishable cell wall between infected cells. Bars (**A**–**C**) = 50 µm, (**D**–**I**) = 5 µm.

Treatment with TMTD at 0.4 mg/kg did not affect the histological structure of root nodules of all studied genotypes, compared with control nodules (Figure 4A–C). Meristematic cells had folded borders (Figure 4D–F); in the laboratory line Sprint-2, numerous small vacuoles merged into large vacuoles (Figure 4D). In the laboratory line SGE, the cell walls of meristematic cells were often cleared (Figure 4E). In the cultivar ‘Finale’, dark inclusions, presumably phenolic compounds, were present in the vacuoles of meristematic cells, and the cell walls were cleared (Figure 4F). The nitrogen-fixation zone contained increased numbers of starch granules (Figure 4G–I). In infected cells, in addition to the central vacuole, many small vacuole-like structures were present, and probably formed due to the expansion of the peribacteroid space in some symbiosomes. This was especially noticeable in the laboratory line SGE and the cultivar ‘Finale’ (Figure 4H,I). In addition, the boundaries between infected cells were barely visible (Figure 4H,I).

**Figure 5:**
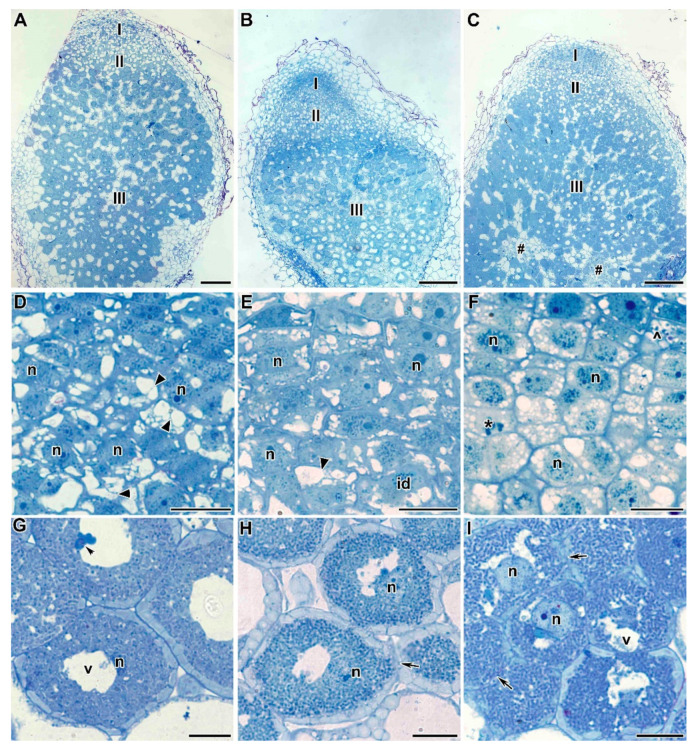
Light microscopy micrographs of 3-week-old plants of three pea genotypes treated with 4 g/kg of TMTD. (**A**–**C**) Longitudinal sections of 3-week-old pea nodules. (**D**–**F**) Meristem of pea nodules. (**G**–**I**) Infected cells in nitrogen-fixation zone of a nodule. Laboratory line Sprint-2 (**A**,**D**,**G**), laboratory line SGE (**B**,**E**,**H**), cultivar ‘Finale’ (**C**,**F**,**I**). **I**—meristem, **II**—infection zone, **III**—nitrogen fixation zone, **n**—nucleus, **id**—infection droplet, **v**—vacuole, *—metaphase plate, **^**—vacuole inclusions, **#**—degenerating cells, arrows indicate the barely distinguishable cell wall between infected cells, arrowhead indicates infection thread with bacteria free outgrowths, triangles indicate vacuoles fusion. Bars (**A**–**C**) = 50 µm, (**D**–**I**) = 5 µm.

At a TMTD concentration of 4 mg/kg, the histological structure of root nodules was similar to that of control nodules (Figure 5A–C), and the meristem, the infection zone, and the nitrogen-fixation zone were visible. In the cultivar ‘Finale’, the nitrogen-fixation zone contained numerous degenerating cells (Figure 5C). Meristematic cells had a folded surface (Figure 5D,E), fused vacuoles (Figure 5D,E), cleared cytoplasm, and dark inclusions in the vacuoles (Figure 5F). There were significantly increased numbers of starch granules in the nitrogen-fixation zone in infected cells (Figure 5G–I). In the laboratory line Sprint-2, the cytoplasm was dense, and there were many large inclusions in bacteroids and many small vacuole-like structures, probably due to the expansion of the peribacteroid space in some symbiosomes. In addition, infection threads formed bacteria-free outgrowths (Figure 5G). In the laboratory line SGE, inclusions were also present in the bacteroids and the tonoplast was disrupted in some places (Figure 5H). In the cultivar ‘Finale’, there were small vacuole-like structures, a disrupted tonoplast, and the nuclei were less dense (Figure 5I). In some cases, it was difficult to distinguish the boundaries between infected cells (Figure 5H,I).

**Figure 6:**
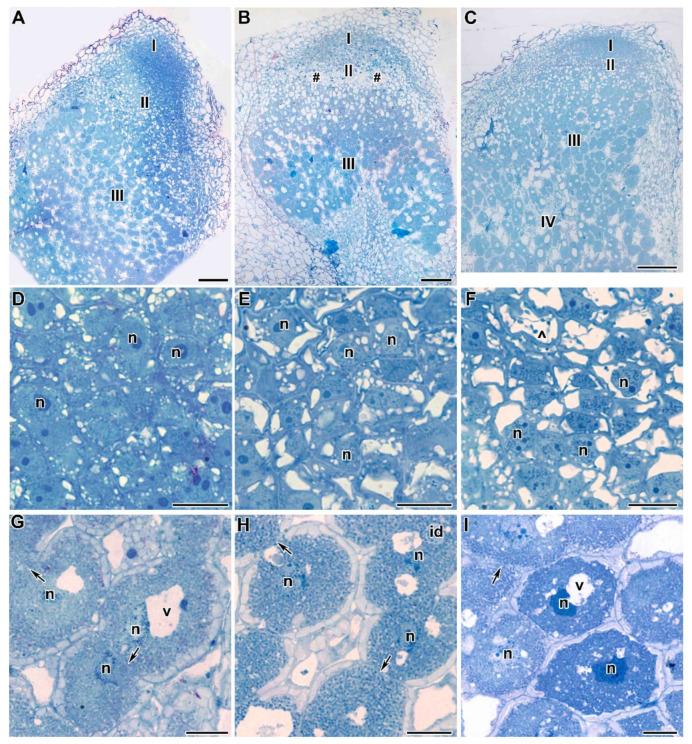
Light microscopy micrographs of 3-week-old plants of three pea genotypes treated with 8 g/kg of TMTD. (**A**–**C**) Longitudinal sections of 3-week-old pea nodules. (**D**–**F**) Meristem of pea nodules. (**G**–**I**) Infected cells in the nitrogen-fixation zone of a nodule. Laboratory line Sprint-2 (**A**,**D**,**G**), laboratory line SGE (**B**,**E**,**H**), cultivar ‘Finale’ (**C**,**F**,**I**). **I**—meristem, **II**—infection zone, **III**—nitrogen fixation zone, **IV**—senescence zone, **n**—nucleus, **id**—infection droplet, **v**—vacuole, **^**—vacuole inclusions, **#**—degenerating cells, arrows indicate the barely distinguishable cell wall between infected cells. Bars (**A**–**C**) = 50 µm, (**D**–**I**) = 5 µm.

At a TMTD concentration of 8 mg/kg, the histological structure of root nodules of the laboratory line SGE and the cultivar ‘Finale’ was altered (Figure 6B,C). Numerous degenerating cells were present in the infection zone and a senescence zone was distinguishable at the base of the nodule (Figure 6B,C). Meristematic cells had a folded cell surface (Figure 6E,F) and indistinguishable cell boundaries (Figure 6D–F), small vacuoles were fused into larger ones (Figure 6E,F), and large inclusions were present in the vacuoles in the cultivar ‘Finale’ (Figure 6F). Starch granules had accumulated in the nitrogen-fixation zone, small vacuole-like structures and large inclusions were present in the bacteroids (Figure 6G–I), and the tonoplast was disrupted (Figure 6H,I). In the cultivar ‘Finale’, infected cells often contained pyknotic nuclei (Figure 6I). In all genotypes, it was difficult to distinguish cell boundaries and cell walls (Figure 6G–I).

Thus, more signs of degeneration, especially disruption of the cell walls, were observed with increasing concentrations of TMTD. Among the three studied genotypes, ‘Finale’ was the most sensitive to TMTD.

### 2.3. Ultrastructure of Nodules

A comparative analysis of the nodule ultrastructure of three pea genotypes was performed. Nodules of three-week-old plants of the laboratory lines SGE and Sprint-2 and the cultivar ‘Finale’ grown without TMTD showed a similar ultrastructural organization, typical of indeterminate nodules. The meristem cells were small and contained electron-dense cytoplasm and numerous small vacuoles (Figure 3D). Cells in the metaphase were frequently observed (Figure 3D). The infection zone contained numerous infection threads and droplets, and several juvenile bacteroids were present in a narrow layer of the cytoplasm around a large vacuole in the cell center (Figure 3E). The nitrogen-fixation zone contained numerous symbiosomes harboring a single pleiomorphic bacteroid (Figure 3F).

Treatment with TMTD caused significant changes in the nodule ultrastructure of all pea genotypes, and the extent of the changes depended on the TMTD concentration and the pea genotype. Structural abnormalities in nodules caused by TMTD were the least severe in Sprint-2 and the most severe in ‘Finale’.

**Figure 7:**
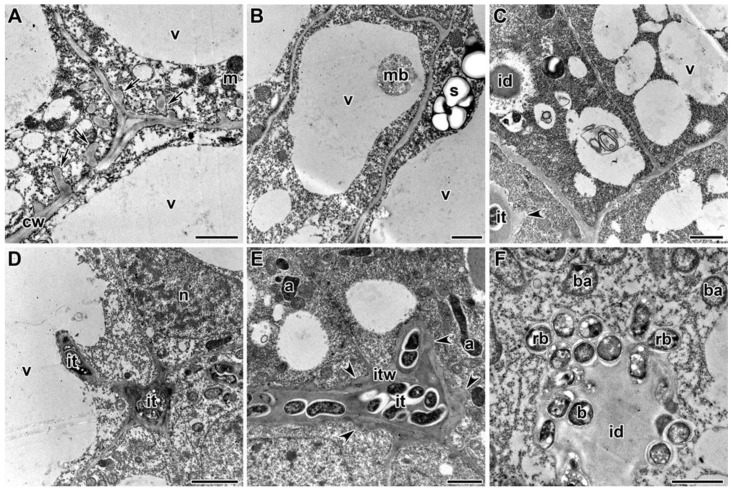
Ultrastructural organization of nodules of pea laboratory line Sprint-2. (**A**) Outgrowths of the cell wall. (**B**) Multivesicular body in a vacuole. (**C**) Electron-translucent swollen cell wall in meristematic cells. (**D**) Infection threads with thickened, swollen walls. (**E**) Thickened infection thread with lateral bulges. (**F**) Unaffected infection droplet. Treated with 0.4 g/kg of TMTD (**F**), treated with 4 g/kg of TMTD (**A**,**B**,**D**), treated with 8 g/kg of TMTD (**C**,**E**). **n**—nucleus, **v**—vacuole, **m**—mitochondrion, **a**—amyloplast, **s**—starch, **cw**—cell wall, **mb**—multivesicular body, **it**—infection thread, **itw**—infection thread wall, **id**—infection droplet, **b**—bacterium, **rb**—released bacterium, **ba**—bacteroid; arrows indicate outgrowths of the infection thread; arrowheads indicate lateral bulges of infection thread walls. Bars (**A**,**E**,**F**) = 1 µm, (**B**–**D**) = 2 µm.

In Sprint-2 treated with TMTD at 0.4 g/kg, the cell ultrastructure did not differ from that of the control at the nodule meristem (data not shown) and the infection zone (Figure 7F). At a TMTD concentration of 4 g/kg, the cell walls in the meristem were less electron dense. The cell wall occasionally grew into the cytoplasm (Figure 7A). Some vacuoles contained multivesicular bodies (Figure 7B). As the TMTD concentration increased to 8 g/kg, the electron density of the cell walls further decreased and swelling of the meristematic cell walls became more pronounced (Figure 7C). The profiles of infection threads in TMTD-treated nodules were morphologically altered. In the infection zone of nodules in the 4 g/kg of TMTD treatment, the walls of the infection threads were thickened and swollen (Figure 7D). In the 8 g/kg of TMTD treatment, the thickened walls of the infection threads in the infection zone of nodules consisted of many layers of fibrous material. The walls often had lateral bulges, of which some were less electron dense (Figure 7C,E). The infection droplets and rhizobia within the droplets were unaffected by TMTD treatment (Figure 7F).

**Figure 8:**
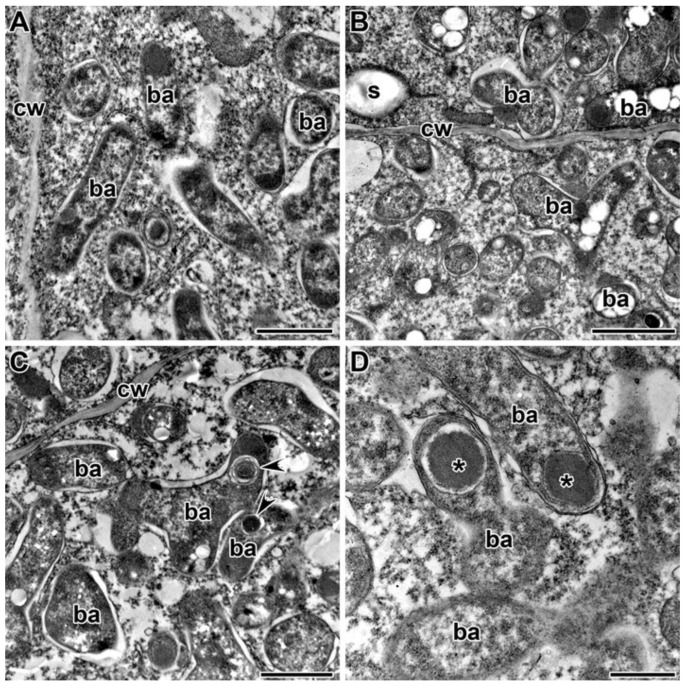
Ultrastructural organization of infected cells in nitrogen-fixation zone of nodules of pea laboratory line Sprint-2. (**A**) Unchanged bacteroids. (**B**) Bacteroids with polyhydroxybutyrate granules. (**C**) Myelin-like inclusions in bacteroids. (**D**) Spherical inclusions with average electron density in bacteroids. Treated with 0.4 g/kg of TMTD (**A**,**B**), treated with 4 g/kg of TMTD (**C**), treated with 8 g/kg of TMTD (**D**). **cw**—cell wall, **s**—starch granule, **ba**—bacteroid, *—spherical inclusion of average electron density; arrowheads indicate myelin-like inclusions. Bars (**A**–**C**) = 1 µm, (**D**) = 500 nm.

Under treatment with 0.4 g/kg of TMTD, some bacteroids in the nitrogen-fixation zone of infected cells were unaffected (Figure 8A) and some contained polyhydroxybutyrate (PHB) granules (Figure 8B). A few bacteroids contained myelin-like structures (Figure 8C) in the 4 g/kg of TMTD treatment. Spherical inclusions with average electron density formed in bacteroids in the 8 g/kg of TMTD treatment (Figure 8D). Thus, in Sprint-2, exposure to TMTD resulted in a loss of electron density and swelling of the cell walls, thickening of infection thread walls, outgrowth formation, and accumulation of PHB and spherical inclusions of unknown nature in the bacteroids.

**Figure 9:**
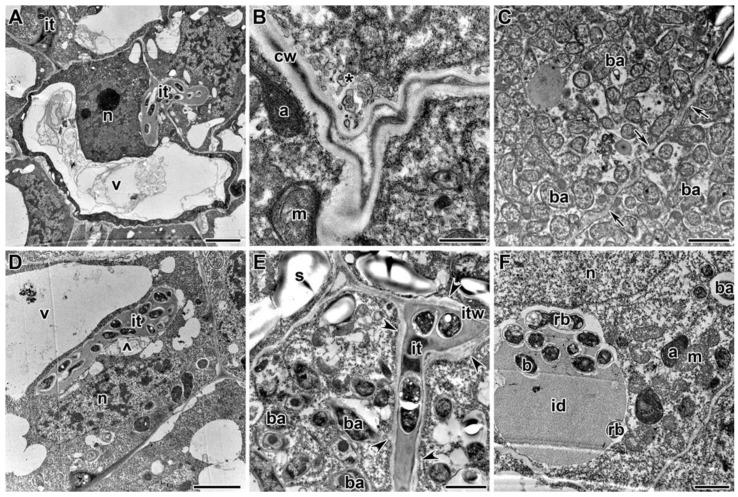
Ultrastructural organization of nodules of pea laboratory line SGE. (**A**) Meristematic cell containing a large vacuole and an infection thread. (**B**) Numerous invaginations and vesicles in plasma membrane. (**C**) Barely distinguishable cell wall between infected cells. (**D**) Numerous vesicles near the infection thread wall. (**E**) Infection thread in an infected cell in the infection zone. (**F**) Infection droplet with released rhizobia. Treated with 0.4 g/kg of TMTD (**D**,**F**), treated with 4 g/kg of TMTD (**A**–**C**), treated with 8 g/kg of TMTD (**E**). **n**—nucleus, **v**—vacuole, **cw**—cell wall, **m**—mitochondrion, **a**—amyloplast, **s**—starch granule, **it**—infection thread, **id**—infection droplet, **b**—bacterium, **rb**—released bacterium, **ba**—bacteroid, *—numerous invaginations and vesicles of plasma membrane, **^**—numerous invaginations and vesicles of plasma membrane near the infection thread; arrows indicate the cell wall; arrowheads indicate lateral bulges of the infection thread wall. Bars (**A**) = 5 µm, (**B**) = 500 nm, (**C**,**D**) = 2 µm, (**E**,**F**) = 1 µm.

In the SGE line treated with TMTD at 0.4 g/kg, cells in the nodule meristem often had an irregular shape with electron-translucent cell walls (data not shown). In response to TMTD at 4 g/kg, the meristematic cells became irregularly shaped with a folded cell wall. In addition, these cell walls were electron translucent and swollen. Numerous small vacuoles characteristic of meristematic cells occasionally merged to form a large vacuole (Figure 9A). The plasma membrane had numerous invaginations, and many vesicles of different sizes formed near the plant cell walls (Figure 9B). The plant cell walls became so thin that the boundaries between cells were barely distinguishable (Figure 9C).

In the infection zone, numerous vesicles proximal to the infection thread wall were engaged in deposition of polysaccharide material at the cell walls through exocytosis (Figure 9D). In response to TMTD at 8 g/kg, the infection threads in the nodules formed thickened walls comprising many layers of fibrous material and often had lateral bulges (Figure 9E). In addition to these changes, spherical inclusions formed in the bacteroids (Figure 9E). In the nodules of the SGE line, the infection droplets were essentially unchanged, but the shape and matrix density of the released bacteria were affected, even by the lowest TMTD concentration of 0.4 g/kg (Figure 9F).

**Figure 10:**
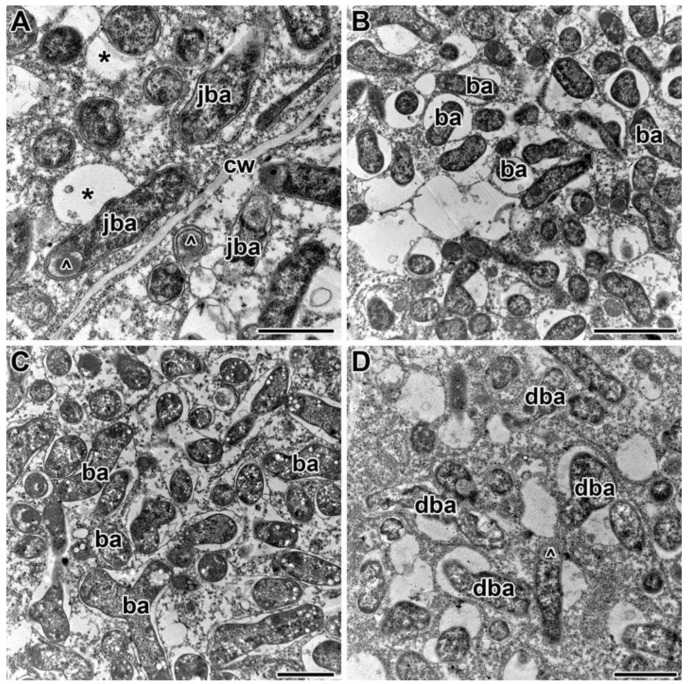
Ultrastructural organization of infected cells from infection and nitrogen-fixation zones of nodules of pea laboratory line SGE. (**A**) Juvenile bacteroids with symbiosome membrane protrusions. (**B**) Bacteroids with enlarged peribacteroid spaces. (**C**) Bacteroids with polyhydroxybutyrate granules. (**D**) Degenerating bacteroids with a matrix with irregular electron density and altered shape. Treated with 0.4 g/kg of TMTD (**A**,**B**), treated with 4 g/kg of TMTD (**C**), treated with 8 g/kg of TMTD (**D**). **cw**—cell wall, **ba**—bacteroid, **jba**—juvenile bacteroid, **dba**—degenerating bacteroid, **^**—spherical inclusions with average electron density, *—protrusions of symbiosome membrane. Bars (**A**,**C**,**D**) = 1 µm, (**B**) = 2 µm.

In response to the lowest TMTD concentration (0.4 g/kg), protrusions of the symbiosome membranes developed, and spherical inclusions of moderate electron density formed in juvenile bacteroids in the infection zone (Figure 10A). In the nitrogen-fixation zone, the peribacteroid spaces became enlarged (Figure 10B). Treatment with TMTD at 4 g/kg caused PHB to appear in the bacteroids (Figure 10C). Plasmolysis of the cytoplasm with a clear pattern of endoplasmic reticulum strands occurred (data not shown). In response to treatment with TMTD at 8 g/kg, bacteroids in the infection zone of infected cells showed initial signs of degradation in the form of an irregular matrix, a folded surface, and numerous vesicles in the peribacteroid space (Figure 10D). Thus, in the laboratory line SGE, TMTD caused not only changes in the plant cell walls and the walls of infection threads, but also degenerative changes in bacteroids in infected cells. The degenerative changes included protrusions of the symbiosome membrane in juvenile bacteroids, expansion of the peribacteroid space, and changes in bacteroid shape and the electron density of the matrix.

**Figure 11:**
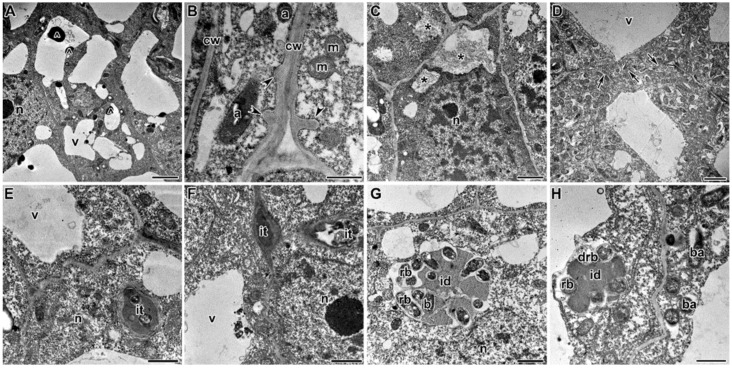
Ultrastructural organization of nodules of pea cultivar ‘Finale’. (**A**) Meristematic cell with electron-dense inclusions in vacuoles. (**B**) Outgrowths of plant cell wall into cytoplasm. (**C**) Numerous invaginations and vesicles of plasma membrane. (**D**) Barely distinguishable cell wall between infected cells. (**E**) Infection thread with thickened wall in the meristematic cell. (**F**) Infection thread with cleared matrix and degenerated rhizobia. (**G**) Infection droplet with fibrillar material within the matrix. (**H**) Infection droplet with released degenerated bacteria. Treated with 4 g/kg of TMTD (**G**), treated with 8 g/kg of TMTD (**A**–**F**,**H**). **n**—nucleus, **v**—vacuole, **cw**—cell wall, **m**—mitochondrion, **a**—amyloplast, **it**—infection thread, **id**—infection droplet, **b**—bacterium, **rb**—released bacterium, **drb**—degenerating released bacterium, **ba**—bacteroid, *—numerous invaginations and vesicles of the plasma membrane, **^**—electron-dense inclusion; arrows indicate the cell wall; arrowheads indicate outgrowths of the cell wall. Bars (**A**,**C**,**D**) = 5 µm, (**B**) = 500 nm, (**E**–**H**) = 1 µm.

In the cultivar ‘Finale’ treated with TMTD at 0.4 and 4 g/kg, the meristematic cells often had an irregular shape with an electron-translucent cell wall (data not shown). Treatment with TMTD at 8 g/kg resulted in cell wall disorders (Figure 11A). Electron-dense inclusions formed in the vacuoles of meristematic cells in plants treated with TMTD at 4 and 8 g/kg (Figure 11A). The cell wall occasionally grew into the cytoplasm (Figure 11B). Some plasma membranes formed numerous invaginations and vesicles that led to the appearance of multivesicular bodies of different sizes near the cell wall (Figure 11C). The thickness of the plant cell wall decreased and the boundaries between cells became barely visible (Figure 11D).

Electron micrographs revealed striking differences in infection threads between untreated and treated nodules, especially in the 8 g/kg of TMTD treatment. The walls of the infection threads in TMTD-treated plants were swollen and became thickened. The thick walls of the infection threads consisted of many layers of fibrous material (Figure 11E). Some infection threads contained an extremely electron-translucent matrix and showed signs of rhizobial degeneration inside (Figure 11F). An abundant fibrillar matrix surrounded bacteria within the infection droplets (Figure 11G). The release of bacteria from infection droplets was impaired and multiple releases of bacteria were observed (Figure 11G). In addition to these changes, newly released bacteria from infection droplets were lysed (Figure 11H).

**Figure 12:**
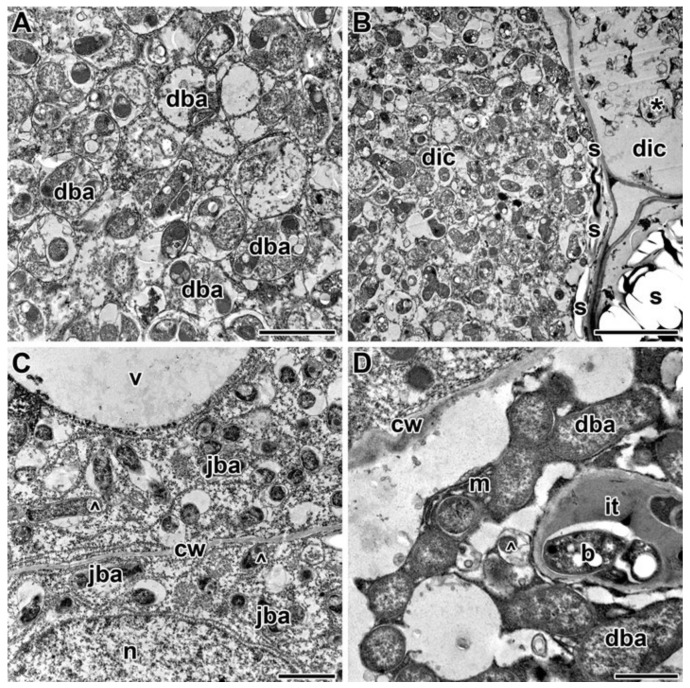
Ultrastructural organization of infected cells in infection zone and nitrogen-fixation zone of nodules of pea cultivar ‘Finale’. (**A**) Degenerating bacteroid with fused symbiosomes. (**B**) Infected cells with degenerating bacteroids and “ghost” bacteroids. (**C**) Juvenile bacteroids with symbiosome membrane protrusions. (**D**) Senescent infected cells with electron-dense cytoplasm. Treated with 0.4 g/kg of TMTD (**A**,**B**), treated with 4 g/kg of TMTD (**C**), treated with 8 g/kg of TMTD (**D**). **dic**—degenerating infected cell, **n**—nucleus, **v**—vacuole, **cw**—cell wall, **m**—mitochondrion, **s**—starch granule, **jba**—juvenile bacteroid, **dba**—degenerating bacteroid, *—“ghost” bacteroid, **^**—spherical inclusions with average electron density. Bars (**A**,**C**) = 2 µm, (**B**) = 5 µm, (**D**) = 500 nm.

Treatment with TMTD at 0.4 g/kg resulted in marked changes in the nitrogen-fixation zone of infected cells. In some cells, as a result of symbiosome membrane fusion, symbiosomes contained several bacteroids at different stages of degeneration (Figure 12A,B). In addition, some infected cells contained “ghost” bacteroids (Figure 12B). Treatment with TMTD caused PHB to appear in the bacteroids (Figure 12A,B). In response to TMTD at 4 g/kg, symbiosome membranes protruded and spherical inclusions appeared in juvenile bacteroids in the infection zone (Figure 12C). In addition, cytoplasm degradation in the form of plasmolysis occurred in the infection zone (data not shown). In the 8 g/kg of TMTD treatment, some senescent infected cells contained aggregated cytoplasmic structures (Figure 12D). Treatment of the cultivar ‘Finale’ with TMTD led to degradation of bacteroids, characterized by expansion of the peribacteroid space, fusion of symbiosome membranes and formation of “multiple” symbiosomes, appearance of “ghost” bacteroids, and premature senescence of infection structures.

Treatment with TMTD affected the plant cell walls and walls of infection threads in each of the studied pea genotypes. However, TMTD did not affect the structure of plastids and mitochondria (Figure 7A,E or Figure 9B,F or Figure 11B). Application of TMTD led to earlier and more abundant accumulation of starch, which is an indicator of an ineffective symbiosis (Figure 7B or Figure 8B or Figure 9E or Figure 12B). Treatment with TMTD reduced the electron density of the cytoplasm in cells of the nitrogen-fixation zone with clear symptoms of plasmolysis (data not shown).

## 3. Discussion

Treatment of legume seeds with fungicides prevents fungal spore germination and reduces mycelial growth, thereby promoting seed germination and seedling growth [23,29,30,32]. Under certain conditions, some fungicides exhibit a phytotoxic effect when applied to legumes. Treatment with different classes of pesticides has been shown to cause growth inhibition, chlorosis, and losses in seed viability in various legume species [27,28,31,33,34,35,36,37,38]. High concentrations of TMTD (>100 ppm) have a deleterious effect on nitrogen fixation by plants. This may be due to the accumulation of ammonia nitrogen or to nitrogen deficiency [30]. In this study, treatment of pea seeds with TMTD resulted in suppressed seedling growth (Figure 1), as well as disruption of nodule formation, especially when high concentrations of TMTD were added directly to the substrate (Figure 2).

Numerous studies have demonstrated the toxicity of diverse fungicides, including TMTD, to rhizobia [15,16,17,18,19,20]. Negative effects of TMTD on nodulation and the nitrogen-fixing ability of nodules of various legumes have been demonstrated previously [24]. Fungicide application has been shown to directly affect nodulation, as opposed to affecting the rhizospheric N_2_-fixing bacterial community [35,39]. In the present study, we observed a toxic effect of TMTD on *Rhizobium*-infected cells in pea symbiotic nodules.

Dithiocarbamic acid and its derivatives, including TMTD, have indiscriminate (multifarious) effects and disrupt a variety of biochemical processes that involve enzymes containing copper or sulfhydryl groups in pathogenic organisms [12,40,41]. The cytotoxic effect of TMTD results from the oxidation of the thiol group of peptides and proteins by the TMTD disulfide group, resulting in the formation of complexes with metal ions or interactions with essential cellular molecules [42,43]. TMTD is metabolized in the organism to form carbon disulfide and dimethyldithiocarbamate, which are also cytotoxic [44]. Their toxicity is due to the formation of reactive oxygen species under oxidative stress, peroxidation of membrane lipids, and mitochondrial dysfunction in cells [43,45].

In another study, the alfalfa (*Medicago sativa* L.) cultivar ‘Apollo’ showed a different reaction to various TMTD concentrations. Treatment with TMTD at low concentrations (3–20 μg/mL) increased nodulation and nitrogenase activity, whereas higher concentrations inhibited these processes [46]. In the present study, we observed genotypic variation in pea responses to TMTD exposure. The cultivar ‘Finale’ was the most sensitive to TMTD treatment and the laboratory line Sprint-2 was the least sensitive. Another study detected genotypic variation in the tolerance of alfalfa cultivars to the fungicide pentachloronitrobenzene [47].

Despite significant negative effects of TMTD on nodulation, its effects on nodule structure have not been described previously. In the present study, the minimum concentration of TMTD (0.4 g/kg) was sufficient to impair the development of symbiotic nodules. This was accompanied by abnormalities in the infection process and premature degeneration of infected cells in nodules. The most obvious structural changes in nodules of ‘Finale’ were impaired growth of infection threads, modification of cell walls and walls of infection threads, and degradation of bacteroids (Figure 12A,B). Treatment with TMTD at concentrations of 4 g/kg and 8 g/kg increased the severity of these disruptions. The latter concentration also changed the histological structure of nodules (Figure 6B,C). A senescence zone formed in the nodules of the TMTD-sensitive genotype (Figure 6C). Other studies found that the initiation of premature senescence of pea nodules occurred in darkness and after treatment with exogenous nitrates [48] and cadmium [49,50].

In this study, treatment with TMTD modified the walls of plant cells and infection threads (Figure 7A,C–E or Figure 8A–E or Figure 11B–F). Despite or perhaps because of their complexity [51,52,53,54], plant cell walls show remarkable adaptive capabilities. The cell walls participate in intercellular communication and protect the cell from biotic and abiotic stresses [53,55,56].

In the present study, the infection threads containing a fibrillar matrix had thicker walls in TMTD-treated plants than in control plants (Figure 7D,E or Figure 9D,E or Figure 11E,F). Similar modifications of the walls and matrix of infection threads have been described for *P. sativum* symbiotic mutants [57,58,59,60,61,62], *M. truncatula* nodules formed by *Ensifer meliloti* mutants with succinoglucan deficiency (EPSI) [63], and *P. sativum* [64] and *M. sativa* nodules [65] formed by lipopolysaccharide-defective rhizobial mutants.

The thickening of cell walls has been described as a response to the action of heavy metals on plants [66,67,68] and on symbiotic nodules in particular [69]. The thickening of the walls can inhibit the penetration of infection threads into nodules and prevent the release of rhizobia from infection threads and droplets [70]. In some plants, the ability of the cell walls to bind metals is increased by the accumulation of polysaccharides such as pectins [71,72].

In the current study, the walls of plant cells and infection threads showed different changes in response to TMTD treatment (Figure 7A,C–E or Figure 8A–E or Figure 11B–F). The walls of infection threads were thickened and formed lateral bulges (Figure 7D,E or Figure 9D,E or Figure 11E,F), while those of plant cells, especially meristematic cells, became thinner, bleached, and deformed (Figure 4E or Figure 9C or Figure 11D). Occasionally, the walls between cells became barely distinguishable (Figure 4H,I or Figure 5H,I or Figure 6G–I). It is likely that an additional mechanism modified the cell wall composition. Similar changes to cell walls, i.e., thinning and deformation, have been observed in response to herbicides that inhibit cellulose biosynthesis [73,74].

Light and electron microscopy analyses revealed inclusions, presumably consisting of phenolic compounds, in vacuoles of meristematic cells in the nodules of the TMTD-sensitive pea cultivar ‘Finale’ (Figure 4F or Figure 5F or Figure 6F or Figure 11A). Phenolic compounds are important biomarkers of xenobiotic detoxification [75,76]. A previous study showed that the use of benzo-(1,2,3)-thiadiazole-7 resulted in the deposition of phenol-rich occlusal material and the formation of structural barriers that prevented the pathogen *Pythium ultimum* Trow from entering the vascular stele of cucumber plants [77]. Elevated contents of phenolic compounds have been reported in several plant species under stress, including wheat under nickel stress [78], corn under aluminum stress [79], faba beans under cadmium stress, and *Phyllanthus tenellus* under copper stress [78]. In *Lotus corniculatus* L. growing on ultramafic soils contaminated with nickel, cobalt, and chromium, vacuoles in the nodule endoderm and some cortical and parenchymal cells were filled with dark phenolic substances [69]. Induction of the synthesis of phenolic compounds in nodules of *M. sativa* plants was observed in the presence of arsenic [80]. Similarly, after inoculation of metal-resistant strains of *M. sativa*, a gene encoding phenylalanine-ammonia lyase, which catalyzes the initial step in lignin synthesis, was expressed in nodules [81]. Previous studies have shown that the synthesis of phenolic compounds is activated not only by xenobiotics, but also in response to an ineffective symbiosis. For example, in the pea mutant SGEFix^–^-2 harboring the allele *sym33-3* of the gene *PsCYCLOPS/PsIPD3*, the release of rhizobia from infection droplets stimulated cell wall formation and suberin accumulation in the vacuole [82].

In this study, we observed accumulation of PHB in bacteroids (Figure 8B,D or Figure 10C or Figure 12A,B). Other studies have shown that the synthesis of PHB is blocked in bacteroids in indeterminate nodules [83,84,85,86,87,88]. In peas, PHB was found to accumulate in the initial stages of infection, but then disappear after bacteria are released and differentiate into bacteroids. Mature bacteroids are noticeably devoid of visible PHB granules [83,84]. Polyhydroxybutyrate protects cells from diverse stresses, including heat shock, ultraviolet radiation, oxidizing agents, and osmotic shock [84]. Previously, we observed accumulation of PHB in bacteroids in response to cadmium exposure [49,50].

Treatment with TMTD also caused the appearance of large spherical inclusions (Figure 5G,H or Figure 6G–I) with moderate electron density in bacteroids (Figure 8D or Figure 9E or Figure 10A,D or Figure 12C,D). Previously, similar inclusions were observed in bacteroids of the ineffective mutant SGEFix^–^-3 (*sym26*), which is characterized by an early nodule senescence phenotype [89]. In the present study, the fusion of symbiosomes and degeneration of bacteroids within them led to the formation of “ghost” bacteroids in the TMTD-sensitive cultivar ‘Finale’ (Figure 12A,B). Similarly, symbiosome fusion and the formation of multiple symbiosomes were found to occur in response to copper exposure in *M. lupulina* nodules [70,90] and cadmium exposure in pea nodules [49,50]. In the present study, we observed the accumulation of PHB in the symbiosomes in bacteroids, bacteroid contraction, an increase in the peribacteroid space, and destruction of symbiosome membranes as a result of lipid peroxidation.

The degradation of bacteria inside infection threads was an additional negative effect observed in nodules of ‘Finale’ plants treated with TMTD (Figure 11F). Previously, we observed degradation of rhizobia inside infection threads as a result of activation of an extremely strong defense reaction in nodules of the pea mutant SGEFix^–^-5 harboring the allele *sym33-2* of the gene *PsCYCLOPS/PsIPD3* [91]. The degradation of bacteria immediately after bacterial release in ‘Finale’ nodules treated with TMTD (Figure 11H) is a striking negative effect of TMTD on nodulation and has not been described previously as a reaction to an ineffective symbiosis.

As a result of exposure to TMTD, the cytoplasm of infected cells became less electron dense and showed clear signs of plasmolysis, including nuclear pyknosis (Figure 6I), but the morphology of the remaining organelles was unaffected. The cytotoxic effect of most xenobiotics, especially heavy metal ions, is manifested as damage to mitochondria as a result of oxidative stress, damage to the plasma membrane, vacuolization of the endoplasmic reticulum and dictiosomes, and damage to nuclei [68,76,92,93,94]. The use of TMTD led to earlier and more abundant formation of starch granules (Figure 4G–I or Figure 5G–I or Figure 6G–I), which is one of the signs of an ineffective symbiosis [89,95]. An interesting effect of TMTD treatment was the formation of multiple meristems (Figure 1B–D). Previously, we observed nodule meristem bifurcation under gibberellic acid (GA_3_) treatment [96].

## 4. Materials and Methods

### 4.1. Plant Material and Bacterial Strain

The pea (*P. sativum* L.) laboratory lines SGE [97] and Sprint-2 [98], and the cultivar ‘Finale’ [99] were used in this study. The streptomycin-resistant *R. leguminosarum* bv. *viciae* strain 3841 was used for inoculation [100]. Bacteria were cultured for 3 days on a solid TY nutrient medium supplemented with streptomycin (600 μg/mL) at 28 °C.

### 4.2. Inoculation and Plant Growth Conditions

Seeds were sterilized with concentrated sulfuric acid for 30 min and then washed 10 times with sterile water. The seeds were germinated in Petri dishes in an incubator (Memmert GmbH, Schwabach, Germany) at 24 °C. For the TMTD treatments, TMTD was added to the vermiculite at a concentration of 0.4, 4, or 8 g/kg, and mixed thoroughly into the vermiculite. Seedlings were transferred to plastic pots containing 150–200 g vermiculite and 250 mL nitrogen-free Fåhraeus nutrient solution [101] and each seedling was inoculated with 1 mL aqueous bacterial suspension (10^7^–10^8^ cells). The plants (15 plants per variety) were grown in a growth chamber (MLR-352H, Sanyo Electric Co., Ltd., Moriguchi, Japan) under the following conditions: 21 °C, light intensity 280 μmol m^−2^ s^−1^, photoperiod 16 h light/8 h dark, and relative humidity 75%. Plants and nodules were harvested at 3 weeks after inoculation.

### 4.3. Growth and Nodulation Parameters

Growth and nodulation parameters were analyzed for the SGE line only. Experiments were performed with 15 TMTD-treated plants and 15 untreated (control) plants. For weight measurements, shoots and roots were separated and cotyledons were removed. Excess moisture was removed from the plants with filter paper. All nodules on the root system were removed with tweezers and counted. Pea nodules were photographed under a SteREO Lumar.V12 stereomicroscope equipped with an AxioCam MRc 5 video camera (Carl Zeiss, München, Germany). Object visualization was performed using AxioVision Rel. 4.8 software (Carl Zeiss).

### 4.4. Statistical Analysis

Statistical analysis of the data was performed using the software STATISTICA version 10 (StatSoft, Inc., Tulsa, OK, USA). Statistically significant differences were evaluated using one-way ANOVA (*p* ≤ 0.05) and the least significant difference test.

### 4.5. Light and Transmission Electron Microscopy Analyses

Nodule ultrastructure was analyzed for all genotypes. About 10–15 nodules were harvested from different plants of each variety and placed directly into fixative. A lateral cut was made on each nodule to improve penetration of the fixative. The nodules were fixed in 2.5% glutaraldehyde (Sigma-Aldrich, St Louis, MO, USA) in 0.01% PBS (2.48 g/L NaH_2_PO_4_, 21.36 g/L Na_2_HPO_4_, and 87.66 g/L NaCl, pH 7.2). Then, the samples were placed under vacuum to remove air from the intercellular spaces and were left in fixative overnight at 4 °C.

The nodules were washed in buffer (four times for 15 min each) and postfixed in 2% osmium tetroxide in 0.1 M phosphate buffer for 2 h. The samples were dehydrated as described previously [88]. Dehydrated samples were progressively infiltrated with Eponate 12 (Ted Pella, Inc., Redding, CA, USA) at room temperature. Finally, samples were embedded in fresh Eponate 12 resin, which was polymerized at 60 °C for 48 h in small plastic containers.

For light microscopy, the embedded material was cut into semi-thin sections (1 μm) using a Leica EM UC7 ultramicrotome (Leica Microsystems, Vienna, Austria). Sections were placed on glass slides (SuperFrost) (Menzel-Gläser, Thermo Fisher Scientific, Waltham, MA, USA) and stained with methylene blue-azure II for 20 min at 65 °C [102]. The sections were examined under a microscope (Axio Imager.Z1) (Zeiss) and images were acquired using an Axiocam 506 (Zeiss) digital video camera.

For transmission electron microscopy, ultrathin sections (90–100 nm thick) were cut with a Leica EM UC7 ultramicrotome (Leica Microsystems) and counterstained as described previously [88]. Nodule tissues were examined using a JEM–1200 EM transmission electron microscope (JEOL Ltd., Tokyo, Japan) at 80 kV. Electron micrographs were captured with a Veleta CCD camera (Olympus, Münster, Germany).

## 5. Conclusions

Our results show that pea symbiotic nodules are highly sensitive to the phytotoxic effect of the fungicide TMTD. High concentrations of TMTD in the substrate cause structural changes in the plant cell walls and infection threads, and premature degradation of bacteroids. The plant cell walls and infection threads show different responses to TMTD. Therefore, further investigations should include studies of the mechanism of cell wall modification, and antioxidant defense systems at the morphological, biochemical, and molecular levels. Such analyses will reveal the mechanism of fungicide toxicity in legumes. To the best of our knowledge, this is the first report on the effects of TMTD on the ultrastructure of symbiotic nodules. An enhanced understanding of the adverse effects of agricultural pesticides on legume crops will be useful for developing strategies to use pesticides optimally in agricultural systems. Notably, TMTD has been banned in the European Union because of health and environmental risks.

## Figures and Tables

**Table 1:** Pearson’s correlation coefficients between TMTD treatment and shoot and root fresh weights (*p* < 0.05).

	Number of Nodules	Shoot Fresh Weight	Root Fresh Weight	TMTD
Number of nodules	1			
Shoot fresh weight	0.807	1		
Root fresh weight	0.726	0.825	1	
TMTD	−0.661	−0.505	−0.733	1
